# Applying Red Mud in Cadmium Contamination Remediation: A Scoping Review

**DOI:** 10.3390/toxics12050347

**Published:** 2024-05-08

**Authors:** Jintao Li, Xuwei Li, Matthew Fischel, Xiaochen Lin, Shiqi Zhou, Lei Zhang, Lei Wang, Jiali Yan

**Affiliations:** 1 School of Civil Engineering and Architecture, Chuzhou University, Chuzhou 239000, China; 2 Nanjing Institute of Environmental Science, Ministry of Ecology and Environment of the People’s Republic of China, Nanjing 210042, China; 3 Sustainable Agricultural Systems Laboratory, USDA-Agricultural Research Service, Beltsville, MD 20705, USA; 4 Ecological Environment Bureau of Chuzhou City, Chuzhou 239000, China

**Keywords:** red mud, cadmium, contaminant immobilization, environmental remediation, heavy metal sorption

## Abstract

Red mud is an industrial solid waste rarely utilized and often disposed of in landfills, resulting in resource waste and environmental pollution. However, due to its high pH and abundance of iron and aluminum oxides and hydroxides, red mud has excellent adsorption properties which can effectively remove heavy metals through ion exchange, adsorption, and precipitation. Therefore, red mud is a valuable resource rather than a waste byproduct. In recent years, red mud has been increasingly studied for its potential in wastewater treatment and soil improvement. Red mud can effectively reduce the migration and impact of heavy metals in soils and water bodies. This paper reviews the research results from using red mud to mitigate cadmium pollution in water bodies and soils, discusses the environmental risks of red mud, and proposes key research directions for the future management of red mud in cadmium-contaminated environments.

## 1. Introduction

Red mud is a strongly alkaline byproduct generated from the extraction of alumina from bauxite, which is rich in iron [Fe(III)] oxides [1]. The yield of red mud is affected by the type of bauxite and processing methods. On average, producing 1 t alumina generates about 1.5 t red mud [2]. The annual world alumina yield is estimated to be over 130 million tons, which creates more than 200 million tons of red mud. However, the reuse of red mud is less than 5%, and most existing red mud remains in storage at disposal pits. Because of the high pH (10–12.5) and the content of toxic elements, such as Th, Zr, Cr, Ni, Ce, V, Nd, Mn, La, Sc, and As, the storage of red mud causes a series of environmental issues. These issues include heavy metal contamination in surface water and groundwater, soil salinization, and air pollution [3,4,5,6,7,8]. Thus, increasing the reuse of red mud has garnered significant attention. Red mud can be used as a valuable material instead of treated as solid waste, solving issues around red mud disposal and its associated environmental risks.

Currently, there are several possible utilization pipelines for red mud. First, to recover valuable metals or rare earth metals, especially Fe, Al, Ti, and Sc [9,10,11,12,13]. Second, to prepare building materials (i.e., cement, brick, glass–ceramic, and road base materials) [14,15,16,17]. More recently, the use of red mud in heavy metal remediation has gained attention as a viable reuse strategy [18,19,20]. Red mud is rich in metal oxides and can be used as a sorbent for heavy metals in environmental and engineered systems. Reusing red mud to remove heavy metals mitigates the risks associated with red mud disposal while simultaneously remediating risks from heavy metal contaminants in soil and water. This review focuses on the feasibility of using red mud to remediate cadmium (Cd) contamination from the environment and in engineered wastewater treatment systems.

Cadmium is a widespread environmental contaminant due to geogenic and anthropogenic sources. It is highly toxic to all organisms and poses a serious threat to human health. Human activities such as fuel combustion, mining and smelting of nonferrous metals, industrial manufacturing, and sewage irrigation have caused Cd contamination in water and soil systems. As a class I carcinogen, Cd has been recognized as a world public health hazard, causing a range of ailments, from damage to the kidneys, the respiratory system, and the gastrointestinal tract, and it is associated with several cancers [21]. Red mud has an excellent Cd adsorption capacity due to its porous structure, high alkalinity, and high iron oxide content, which could be used as a Cd immobilizer. Many unknowns remain regarding Cd availability based on the red mud source, the soil type, and the geochemical conditions. This review summarizes the reported studies on using red mud in Cd remediation, the passivation mechanisms and impact factors, and the environmental risks of red mud application. This review synthesizes a scientific basis for Cd remediation with red mud using the current literature and identifies critical areas of future research.

## 2. Red Mud Characterization

### 2.1. Elements and Minerals Constituents

Extracting alumina from bauxite primarily relies on three industrial processes: the Bayer process, the sintering process, and the combination Bayer–sintering process [2]. Among these processes, the Bayer process produces more than 95% of the world’s alumina due to its low energy cost and simple production methodology [3]. Red mud is the byproduct of these processes and primarily contains lime (CaO), aluminum oxide (Al_2_O_3_), iron(III) oxide (Fe_2_O_3_), silica (SiO_2_), titanium dioxide (TiO_2_), and sodium oxide (Na_2_O). Red mud comprises smaller quantities of additional elements, including K, Cr, V, Ba, Mn, Cu, Zn, Pb, P, F, S, and As. The composition of red mud varies with different production processes. For example, Bayer red mud contains more Fe_2_O_3_ and Al_2_O_3_ and less CaO than red mud from the other two processes (Table 1). There are also distinct mineralogies between red muds produced by each process. Red mud created from sintering contains mineral phases such as β-dicalcium silicate (2CaO·SiO_2_), calcite (CaCO_3_), hematite (Fe_2_O_3_), aragonite (CaCO_3_), perovskite (CaTiO_3_), and gibbsite (Al(OH)_3_) [22]. The Bayer red mud contains mineral phases such as gibbsite [Al(OH)_3_], hematite (Fe_2_O_3_), goethite [FeO(OH)], calcite (CaCO_3_), rutile (TiO_2_), and quartz (SiO_2_) [23].

### 2.2. Chemical and Physical Properties

Red mud has a high pH of 10–12.5 and a cation exchange capacity of 43–75 meq 100 g^−1^ [27], indicating an excellent adsorption capacity. The zero-point charge (ZPC) among the components of red mud varies; the pH_zpc_ of SiO_2_ is 2.3; the pH_zpc_ of Fe_2_O_3_ is 8.6; the pH_zpc_ of α-Al_2_O_3_ is 9.2; and the pH_zpc_ of red mud represents a composite of these individual components and varies from 6 to 8.7 [28,29,30,31]. However, it is important to note that depending on the pH, individual components can be above or below their ZPC, which can cause differences in electrostatic sorption to specific components. For example, at pH 9, the Fe_2_O_3_ will have a negative surface charge, while α-Al_2_O_3_ will exhibit a positive surface charge, irrespective of the overall red mud ZPC.

Particle size is an important physical property that influences the chemical properties of a material (Table 2). The particle size of red mud varies from different production processes. Generally, the Bayer red mud has a smaller particle size ranging from 2 to 18 μm [32,33], while the sintering and combined red mud has a particle size typically larger than 10 μm [24]. The particle density of red mud depends on the production temperature and usually ranges from 3 to 3.8 g cm^−3^, with a specific surface area of 15–30 m^2^ g^−1^ [34,35,36]. Red mud has a porous structure characterized by a void ratio ranging from 2.5 to 3.0 [37]. Its pore structure is loose, and the distribution of mineral particle sizes is extensive. The melting point of red mud falls between 1200 and 1500 °C. Upon its initial discharge, the moisture content fluctuates between 82.3 and 105.9%. The saturation level spans from 91.1 to 99.6%. The plasticity index ranges from 17 to 30, while the liquid limit is 1.31 to 1.56 [38,39]. Despite its high porosity and substantial water content, red mud does not undergo shrinkage or expansion upon drying or wetting [37].

## 3. Red Mud Chemical and Physical Properties Impacting Cadmium Remediation

Red mud’s physical and chemical properties make it an ideal sorbent for cadmium in engineered and environmental systems. Red mud can decrease the Cd bioavailability by direct adsorption or changing the physicochemical properties, for instance, enhancing the soil pH. Many studies have confirmed the stabilization of Cd by red mud amendment in both water and soil [40]. For example, Zhu et al. found that granulated red mud could entirely remove low-concentration Cd in water [41], and Lee et al. found that red mud amendments could efficiently stabilize Cd in soil [42]. However, lingering unknowns on the safety of using red mud as a sorbent in wastewater systems and as a soil amendment have limited the use of red mud in real-world applications. The following section synthesizes the current literature characterizing red mud’s chemical and physical properties to provide a basis for applying red mud to remove heavy metals from environmental and engineered systems. Although red mud contains heavy metals, they are mostly unavailable, and the high proportion of metal oxides in red mud makes it an excellent adsorbent for heavy metals like Cd. 

### 3.1. Sorption Mechanisms

**Figure 1:**
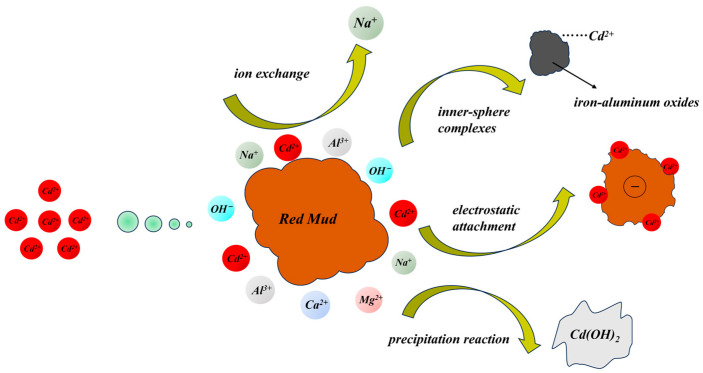
Sorption and passivation mechanisms impacting red mud adsorption of Cd.

The adsorption of Cd^2+^ on red mud includes non-specific adsorption and specific adsorption (Figure 1). Red mud has a large specific surface area, which provides many active adsorption points, leading to fine physical adsorption and the ability to capture Cd^2+^. The porous structure of red mud is conducive to the diffusion of Cd^2+^ to inner sites and provides space for Cd^2+^ adsorption. Cadmium can enter the red mud pores and adsorb by physical embedding [43,44]. Furthermore, the surface of red mud has a negative charge, which generates electrostatic attraction to Cd^2+^ and contributes to Cd^2+^ adsorption [45].

Specific adsorption is the dominant mechanism of Cd adsorption on red mud [46,47]. Adding red mud increases the amounts of -OH and -COOH adsorbed on the soil surface, forming organic Cd complexes, thus decreasing the available Cd in the soil aggregates [48]. Also, high alkalinity adjusts the charge of the repairing agent, creating favorable conditions for ion exchange mechanisms, whereby Cd^2+^ can be adsorbed through ion exchange with the positive charges of the exchangeable site to form outer-sphere complexes [49,50,51,52,53]. In addition, the sodium in the red mud may elevate the soil’s electrical conductivity (EC), leading to the mobilization of heavy metal cations and facilitating their adsorption onto negatively charged soil particles [54]. In a heat treatment red mud study, Yang et al. found that the Cd^2+^ could adsorb onto red mud by forming -OCdOH or partly through metal–metal ion exchange with Na^+^ [55]. Peng et al. found that the mechanisms of Cd^2+^ adsorption on amorphous manganese dioxide-modified red mud could be electrostatic attachment, specific adsorption (i.e., Cd-O or hydroxyl binding), and ion exchange. Furthermore, adding red mud to the soil could enhance the soil pH and transfer the available Cd to hydroxide precipitation, thus decreasing the soil Cd mobility [48,56,57]. 

Red mud contains Fe and Al oxides/hydroxides, which can adsorb Cd and form inner-sphere complexes [55]. These inner-sphere complexes are stable and resist Cd desorption. Metal oxides represent a substantial proportion of the red mud sorption potential. Gibbsite, the predominant Al oxide found in red mud, can bind Cd^2+^ through a bidentate mononuclear bond [58]. The Fe oxides hematite and goethite can electrostatically adsorb Cd and bind Cd through ternary complexes with anions such as phosphorus [59]. Cadmium can also be removed from the matrix by surface precipitation onto these Fe oxides [59]. In addition to the Al and Fe oxides, Ti oxides, like rutile [60], can adsorb Cd electrostatically or by forming ternary complexes [61]. Red mud created by the Bayer process likely has more Cd adsorption potential because of its higher composition of Fe and Al oxides relative to the sintering and combination processes.

### 3.2. Factors Impacting Cadmium Adsorption

(1) pH

pH is a key factor that impacts the effects of red mud on Cd. In acidic environments, H^+^ accumulates on the surface of red mud particles and competes with Cd^2+^ for available sites. Since H^+^ is more likely to exchange ions with the metal cations inside the adsorbents and occupy the majority of the active sites, H^+^ hinders the adsorption of Cd^2+^ on red mud [23,62]. Xu et al. determined that raising the pH of the reaction system significantly enhanced the Cd removal by red mud, with the highest removal rate of 96.2% at pH 9 [48]. Sahu et al. found that the adsorption of Cd^2+^ on HCl-activated red mud was highly pH dependent, with the Cd removal rate increasing with more basic pHs. As pH increases, the Cd available can also be impacted by the formation of Cd carbonate [63]. This phase is less stable to pH change but can alter the availability of Cd for sorption and the fate and transport of Cd in environmental systems.

Another chemical property that governs Cd^2+^ sorption to red mud is the ZPC, which determines the overall surface charge of the red mud composite. When the solution pH is below the ZPC, the surface of the red mud has a positive charge density, leading to a low Cd^2+^ adsorption capacity due to the electrostatic repulsion. Many metal oxides, like goethite and Al_2_O_3_, that are responsible for sorbing Cd within red mud have alkaline ZPCs. With the increase of the pH, the negative charge density of the red mud surface increases, and the adsorption of Cd^2+^ is enhanced [64]. Concurrently, the decrease of H^+^ promotes the ionization of COOH into H^+^ and COO−. The increase of COO− on the surface of red mud promotes the electrostatic attraction of Cd^2+^ and COO− and increases the adsorption of Cd^2+^ [65]. Hydrolysate Cd^2+^ into CdCl^+^ and CdOH^+^ may also contribute to enhancing Cd^2+^ adsorption on red mud due to a higher ion exchange [66]. When the pH > 9, the Cd(OH)_2_ precipitation also promotes Cd^2+^ removal [66].

(2) Coexisting ions

Common metal cations in natural water or sewage, such as Na^+^, K^+^, Ca^2+^, and Mg^2+^, are prone to competitive adsorption with Cd^2+^ [67]. Peng et al. studied the influence of Na^+^, K^+^, Ca^2+^, and Mg^2+^ on Cd removal, and the results showed that these cations competed with Cd^2+^ for limited adsorption sites on the surface of red mud and then inhibited the Cd^2+^ adsorption [44]. Ca^2+^ has a similar electron radius to Cd^2+^, and the relatively small hydrated ionic radius of Ca^2+^ makes it easier to compete with Cd^2+^ for adsorption, so the inhibition effect of Ca^2+^ to Cd^2+^ is the most significant [68,69]. If the coexisting cation has a large electron radius, like Pb^2+^, it can increase the probability of collision with active substances and bolster the adsorption on red mud compared to Cd^2+^ [70]. The positive or negative charges are another critical factor determining the adsorption affinity [71,72]. Cations with higher electronegativity also compete with Cd^2+^ due to a stronger attraction to the red mud particle surface [70]. While removing Cd^2+^ from industrial sewage or natural water, there are other impurities besides the competitive ions, such as fibers, inorganic salts, and pigments from paper production. Therefore, the effect of red mud on Cd removal in different types of waste streams requires further study.

Usually, soil is not only contaminated by Cd but also by other heavy metals. Additional heavy metals can compete for the soil adsorption sites with Cd. However, to our knowledge, no experiments with control variables were conducted to determine the impact of other heavy metals on Cd behavior in soil with red mud amendments. Considering the presence of complex components such as organic matter, clay minerals, and metal oxides in soil, the stability of adsorbents, the differences in adsorption mechanisms between soil and water [73], and whether the conclusions from water-based experiments are relevant to soil applications require further verification and optimization. These considerations will ensure the adsorbent can also exert good heavy metal removal in soils and efficiently treat heavy metal pollution in water and soil.

(3) Temperature

Temperature is another factor that affects the removal of Cd by red mud. The amount of Cd adsorbed by red mud varies from different temperature conditions. Bai et al. studied the influence of temperature on the removal efficiency of Cd^2+^ by red mud and showed increasing temperature promoted the adsorption of Cd^2+^, and the removal rate stabilized after 40 °C [70]. With increasing temperature, the probability of Cd^2+^ colliding with the surface of red mud particles rose and thus increased the reaction rate [74]. Sahu et al. conducted batch experiments at a range of temperatures (10–90 °C) and found that the Cd^2+^ adsorption capacity of red mud gradually increased with the increase in temperature [45]. Yang et al. found that with a low initial Cd concentration, adsorption was independent of temperature, likely caused by sufficient adsorption sites on the red mud surface [66]. Increasing temperature can also cause more sorption because of the higher overall system energy and decrease in solution density, which lessens physical barriers to sorption. Raising temperature also alters minerals’ ZPC and results in a more negative surface charge, which favors Cd adsorption [75].

(4) Ligands

Ligands can impact Cd sorption by red mud by either increasing or decreasing sorption depending on the conditions of the solution matrix. Ligands from low-weight organic compounds are abundant in soils and wastewater from the decomposition of plant material, and these ligands can influence the Cd solubility and sorption potential. At acidic pHs, ligands can serve as bridging complexes and increase adsorption onto mineral surfaces within red mud [76]. However, these ligands can also directly complex the Cd, making it unavailable for sorption. As discussed previously, OH^−^ can impact Cd sorption, and it is also involved in ligand exchange, where it is substituted in the complex between a metal like Fe oxide with Cd in red mud to form a Fe-O-Cd inner-sphere bond [63]. The presence of specific ligands depends on the matrix where the red mud is applied; however, it is clear ligands are an important factor to consider when determining the Cd sorption potential of red mud, especially in the rhizosphere of plant and soil systems. 

Table 3 summarized the sorption mechanisms and factors impacting Cd remediation with red mud.

## 4. Using Red Mud in Cadmium Remediation

### 4.1. Cadmium Remediation in Water

#### 4.1.1. Cadmium Adsorption Capacities of Red Mud

Although many water treatment methods exist, adsorption is still the most widely used. The porous structure of red mud makes it an excellent adsorbent for aqueous Cd removal, and it has the advantages of low cost and simple operation. Table 4 summarizes the adsorption capacity of red mud-based adsorbents for Cd-contaminated sewage. Note that it is usually necessary to activate the red mud (including acid treatment, heat treatment, and neutralization) to improve the adsorption capacity when preparing red mud-based adsorbents [23], as well as prevent secondary pollution caused by harmful components of red mud [77]. In a powdered form, red mud can be challenging to recover from water treatment applications, so many applications pelletize the red mud for ease of use and recovery [78].

In recent years, research on red mud has become increasingly comprehensive, with a shift in focus from single materials to composite materials to enhance the ability of red mud to capture and fix Cd pollutants. Liu et al. found that the maximum Cd^2+^ adsorption capacity of polyacrylic acid-modified red mud was 855 μmol g^−1^, 4.4 times higher than the original red mud [65]. Yang et al. studied the adsorption strength through a simulated rainwater leaching experiment, and they determined the adsorption stability of Cd^2+^ by 500 °C heat-treated red mud was almost double that of the original red mud [55]. Peng et al. prepared manganese dioxide-modified red mud, and they found that it has a coarser surface, larger specific area, and higher pore volume than the original red mud, leading to a three times higher adsorption equilibrium [44].

#### 4.1.2. The Reuse of Red Mud-Based Cd Sorbent

Whether red mud can be used as a potential adsorbent also depends on its desorption performance and reusability. It is necessary to develop a practical adsorbent regeneration method. Compared to powdered red mud, granular red mud adsorption materials offer a solution to the issue of the ease of aggregation, regeneration, and recovery of red mud in the wastewater treatment process. Zhu et al. employed 0.1 M HCl to continuously desorb Cd from granular red mud [41]. Each desorption cycle was within 140 min. The recovered granular red mud could be utilized for up to four cycles before it was exhausted [41]. Peng et al. determined the Cd^2+^ adsorption capacity of the manganese dioxide-modified red mud was reduced by approximately 35% after five adsorption–desorption cycles, showing a good Cd^2+^ adsorption capacity and regeneration ability [44]. The removal rate of the adsorbent exhibits a gradual decline with the increase in the number of cycles. This phenomenon may be attributed to the loss of a minor portion of the adsorbent in each treatment, which reduces the adsorbent mass and the available adsorption sites [81]. Sahu et al. found that the maximum adsorption capacity of Cd^2+^ by acid-activated red mud was 12.6 mg g^−1^, and the regeneration rate reached 77–91% by treating it with 0.1–0.2 M HCl [45]. Khan et al. found that 0.1 M HNO_3_ had the best desorption effect on used iron oxide-activated red mud with an adsorption rate of regenerated red mud for Cd^2+^ up to 91% [80]. They also determined the red mud can be recycled up to five times [80]. These studies prove the feasibility of red mud for Cd-contaminated sewage treatment.

### 4.2. Soil Cadmium Remediation

Red mud’s high alkalinity and strong Cd adsorption capacity make it a candidate for soil Cd remediation. Additionally, red mud contains phosphorus, calcium, and magnesium, which can provide essential nutrients for plant growth [82]. Studies focusing on the effect of red mud on soil Cd speciation and plant Cd concentration are increasingly common.

#### 4.2.1. Impacts of Red Mud on Soil Cadmium Availability

Table 5 summarizes the effects of red mud on soil Cd immobilization. Lee et al. found that applying 2% and 5% red mud lowered the soil available Cd by 89% and 98%, respectively, compared to the control groups [41]. The reduction in Cd availability is likely due to the increase in soil pH with the red mud application. Xu et al. found that OH^−^ released by red mud can neutralize with H^+^ adsorbed on the surfaces of clay minerals, creating additional adsorption points for free Cd^2+^ [48]. Friesl et al. performed a long-term experiment applying red mud, and their results showed applying 1% red mud reduced the soil extractable Cd by 90% [83]. Wang et al. added 3% and 5% red mud to the soil, and the pH of the soil increased by 0.3 and 0.5 units, respectively. When the red mud dosage was 5%, the highest stabilization efficiency of Cd reached 68% [84]. Feng et al. also observed similar results of Cd availability with a red mud application [85]. Pavel et al. conducted a six-year remediation experiment on heavy metal-contaminated soil using red mud. The results demonstrated that red mud reduced the extractable Cd concentration in the soil by 86%. This research provides a feasible solution for the long-term remediation of heavy metal-contaminated soil with red mud [86]. In Friesl et al.’s study, applying 5% red mud reduced the soil extractable Cd by 33%; however, the high As, Cr, and V content may cause harm to soil and crops [87]. 

Red mud can alter soils’ physical and chemical properties, thus changing Cd fate and mobility. Garau et al. found that adding red mud to a sub-acidic soil caused a substantial loss in soil organic matter [90]. They postulate this initial carbon loss could be due to a priming mechanism due to the high sodium content or alkaline pH, which caused the release of the stabilized C and N into the water-extractable pool. This dissolved organic carbon and nitrogen pulse can cause further microbial growth and a positive feedback loop while providing a source of ligands that can alter Cd availability. Red mud can increase soil aggregation, which can impact the structural properties of the soils and potentially alter properties such as the spatial distribution of redox, the formation of anaerobic microsites, and the stability of iron oxides and adsorbed Cd [48]. These studies demonstrate the need for more research on different soil types and in soil and plant systems to understand how red mud alters Cd availability in complex soil systems. Therefore, the amount of red mud used in soil remediation must be adjusted according to both red mud and soil properties. In some cases, it may be necessary to pre-treat the red mud. The effect of red mud on soil Cd remediation depends on the type of soil and red mud, along with the application rate, so preliminary experiments should be conducted to determine the red mud dosage before in-field utilization.

#### 4.2.2. Impacts of Red Mud on Plants Cadmium Accumulation

Red mud can change the soil Cd availability and thus affect plant Cd uptake. In the reported studies, the Cd concentrations in most plants grown on the red mud treated soil were reduced by over 50%, compared to the untreated soil (Table 6). In dry land, applying red mud could reduce Cd bioavailability and decrease Cd content in plants (e.g., spinach, tomato, cabbage, radish, and lettuce) [41,91]. Red mud can increase the biomass and improve the growth of peas and wheat [92]. However, both plants showed symptoms of leaf necrosis after chlorosis, probably due to the binding of Fe and Al oxides in the red mud to phosphorus and other nutrient elements [92]. In the case of paddy fields, Li et al. conducted a one-year field experiment on remediating Cd-contaminated paddy soil with red mud. The results showed that red mud reduced the soil acidity, promoted the shift of exchangeable Cd to other forms, and then reduced the concentrations of Cd in rice roots, husks, and grains [93]. And because the red mud amendment contained Mg, Si, and other beneficial elements needed by crops, the red mud also improved the maturity and yield of rice plants [93]. The application of red mud as amendments in Cd-contaminated soil can effectively reduce the availability of Cd, increase crop growth, and reduce the Cd concentration in the edible parts of plants, which provides an effective way to improve soil quality. 

#### 4.2.3. Impacts of Red Mud on Soil Microbes

The impact of red mud applications on the soil microbiome related to Cd uptake requires further research. Xu et al. determined red mud’s impact on the rhizobacteria and Cd uptake and found that the red mud also reshaped the abundance and composition of the bacteria in the rhizosphere. They found that the red mud application stimulated microbial growth, which decreased Cd availability through chelation and biosorption [96]. Feigl et al. studied the impact of red mud dose rate on the bacterial abundance in a surface soil and subsoil [97]. The red mud increased microbial abundance in the surface soils when up to 20% red mud was applied; however, this effect only lasted ten months, and at application rates from 30 to 50%, the microbiome diversity decreased. A different impact was shown in the subsoil, where red mud application rates from 5 to 20% increased microbial diversity for more than ten months, and only red mud applications of 50% decreased microbial diversity in the subsoil. This study used the Biolog EcoPlate parameters, which Xu et al. state only use the 16S rDNA and is not a complete analysis [96]. These results highlight the need for future studies in diverse soils to determine the long-term impact on the soil microbiome. 

## 5. Environmental Risks

Although red mud can remove targeted pollutants, the alkaline compounds (K_2_O and Na_2_O) and heavy metals in red mud pose an environmental risk [98]. Pang et al. determined red mud had a low leaching toxicity and that the manganese dioxide-modified red mud had even lower heavy metal concentrations in the leachate [44]. The metal concentrations in the leachate were below the limit of China’s drinking water quality standard, indicating its safe use in the environment [44]. Cui et al. tested the red mud leachate and found that the concentration of heavy metals in the leachate did not exceed the solid waste detection standard [73]. Yue et al. determined the heavy metal content in the red mud leaching solution was lower than the standard limit, and the heavy metal adsorbed in red mud was relatively stable [99]. After sewage treatment, the spent red mud can be treated to desorb heavy metals and reused. This research indicates red mud can be safely used for sewage treatment.

When red mud is used in soil remediation, it mixes with the soil matrix and forms a long-term stable system. Therefore, compared with sewage treatment, red mud applied to soil remediation requires stricter environmental safety standards. Ujaczki et al. reported that adding 5% *w*/*w* red mud had no significant adverse effects on the soil ecosystem, and the total metal content in the studied soil did not exceed the limit value of local regulations [100]. On the other hand, the heavy metal(loid)s in red mud mainly exists in a residual fraction and is only released at pH < 2 [101]. Furthermore, the treatment of 5% red mud increased the CaCO_3_ content and soil water holding capacity, improved soil structure, and benefited the growth of the soil microbiome [100]. Garau et al. conducted a two-year study on red mud remediation of soil and found that red mud improved the resistance of microorganisms to metals and the activity of microorganisms in soil [90]. 

On the contrary, Torres-Quiroz et al. employed red mud to facilitate the immobilization of heavy metals within a sand matrix and found conflicting results on the soil heavy metal availability. Incorporating 5% red mud fixed 32% of the zinc and 37% of the copper. However, the administration of elevated doses of red mud resulted in a near doubling of the lead content [102]. In a field experiment, adding 5% red mud increased the total Cr concentration of the soil [89].

The concentration and mobility of the heavy metals contained in red mud are relatively low under alkaline conditions and do not typically pose significant environmental risks [103]. Rubinos et al. employed a continuous extraction method to measure the affective content of heavy metals in red mud, and they found that less than 0.6% of chromium, nickel, lead, and zinc were in exchangeable states [104]. On the other hand, the findings of Kutle et al. indicate that under pH 7 conditions, the exchangeable chromium content in red mud is 0.2%. However, when the pH is reduced to 3.5, this value increases to 48.4%, suggesting that the form of contaminants in red mud can vary with changes in pH [105]. Nevertheless, the effects of environmental changes on the form and desorption behavior of pollutants in red mud have not yet been verified in the practical application of red mud to remediate soil heavy metal contamination. Although the red mud environmental risks are assumed to be low, before application, the red mud selection and application rate should be strictly controlled to ensure environmental and human safety throughout the soil remediation process.

## 6. Future Studies

Due to its unique structure and chemical composition, red mud shows an excellent capacity in Cd adsorption, and existing studies confirmed the possible utilization of red mud in Cd environmental remediation. However, there are still many unresolved questions about the mechanisms for Cd removal and their long-term stability. Further studies should focus on the following aspects of Cd remediation by red mud to fill critical gaps in the literature and our understanding of how to reuse this industrial byproduct for environmental remediation.

(1) The removal mechanism of red mud for Cd is not fully understood, especially the competitive adsorption when multiple contaminants or ligands coexist, which needs further study.
(2) The Cd immobilized by red mud could be released from the soil due to environmental changes. Therefore, long-term field monitoring is needed to study the effects of red mud on soil Cd behavior in the environment, including changes to pH and the impact of siderophores on the long-term stability of Cd in soils with red mud applications.
(3) Although the pollutants in red mud are not likely to be a risk to the environment when red mud is used as a Cd immobilizer, and the pH remains circumneutral or alkaline, the effects of red mud on soil ecology, such as the soil microbiome, nutrient cycling, and other ecosystem functions, require further study.
(4) Most red mud-based adsorbents are fine powders, which causes difficulties in recycling and reuse. Improving the recovery rate of red mud-based adsorbents is also a future research focus.

These four main pillars of future research will further solidify the scientific foundation for the application of red mud in Cd remediation from soils and water. Increasing our understanding of the Cd remediation potential of red mud will provide a scientific basis for red mud reuse with tangible benefits to the health of humans and the environment. 

## Figures and Tables

**Table 1:** The chemical constituents of bauxite residue generated using different production processes. All values are shown in %.

Chemical Constituents	CaO	Al_2_O_3_	Fe_2_O_3_	SiO_2_	TiO_2_	Na_2_O	K_2_O	MgO	Reference
Sintering process	40.2	10.4	8.0	17.3	7.1	3.3	0.05	-	[24]
Combination process	40.8	7.7	11.0	22.7	3.3	2.9	0.38	1.77	[25]
Bayer process	3.10	26.4	30.2	14.4	6.0	5.9	0.25	0.43	[26]

**Table 2:** Properties of red mud.

Parameter	pH	pH_ZPC_	CEC (meq/100 g)	Particle Size (μm)	Particle Density (g cm^−3^)	Specific Surface Area (m^2^ g^−1^)	Melting Point (°C)	Saturation Level (%)	Plasticity Index	Liquid Limit
Red mud	10–12.5	6–8.7	8.0	2–18	3–3.8	15–30	1200–1500	91.1–99.6	17–30	1.31–1.56

**Table 3:** Sorption mechanisms and factors impacting Cd remediation with red mud.

Sorption Mechanisms		Description
Specific adsorption	Electrostatic attachment	The surface of red mud has a negative charge, which can produce electrostatic attraction to Cd^2+^.
	Physical embedding	The porous structure of red mud is conducive to the diffusion of Cd^2+^ into the interior and adsorption through physical embedding.
Non-specific adsorption	Inner-sphere complexes	The iron and aluminum oxides contained in red mud can adsorb Cd and form a stable inner sphere complex.
	Ion exchange	Cd^2+^ can be adsorbed by ion exchange with positive charges at the exchange sites.
	Precipitation reaction	The addition of red mud increases the pH value, which can convert the available Cd into a Cd-hydroxide precipitate.
Factors Impacting Cadmium Adsorption		
pH		In an acidic environment, H^+^ accumulates on the surface of red mud particles and competes with Cd^2+^ for available sites, hindering the adsorption of Cd^2+^ on red mud. Increasing the pH of the reaction system can significantly improve the adsorption of Cd^2+^ on red mud.
Coexisting ions		The coexisting cations in the multi-component reaction system can compete with Cd^2+^ for adsorption sites, resulting in the diminished adsorption capacity of red mud for Cd^2+^ compared to a single adsorption system.
Temperature		Within a certain range, the increase in temperature increases the probability of collisions between Cd^2+^ and the surface of red mud particles, accelerates the reaction rate, and improves the Cd^2+^ adsorption capacity of red mud.
Ligands		Ligands can impact Cd sorption by red mud by either increasing or decreasing sorption depending on the conditions of the solution matrix.

**Table 4:** Adsorption capacities of red mud-based adsorbents for Cd-contaminated sewage.

Absorbents	Sorbent Dose (g·L^−1^)	Initial Cd^2+^ Concentration (mg·L^−1^)	Temperature (°C)	Equilibrium Time (h)	pH	Maximum Sorption (μmol·g^−1^)	Reference
Fly ash-modified red mud	2	300	25	1.5	6	1105	[62]
Manganese dioxide-modified red mud	1	50	25	24	6	922	[44]
Polyacrylic acid-modified red mud	1.25 × 10^−3^	100	25	5	6	855	[65]
Red mud heated at 500 °C	0.5	200	40	24	6	674	[66]
Red mud heated at 500 °C	0.5	200	20	24	9	491	[66]
Red mud heated at 500 °C	0.5	200	30	24	6	447	[66]
Red mud heated at 500 °C	0.5	200	20	24	7	397	[66]
Red mud heated at 500 °C	0.5	200	20	24	6	380	[66]
Red mud	0.5	200	20	24	6	286	[55]
Ball-milling nano-particle red mud	25	1124	25	48	6.5	210	[79]
Red mud	1.25 × 10^−3^	100	25	5	6	193	[65]
Acidified red mud	25	1124	25	48	6.5	190	[79]
Red mud	25	1124	25	48	6.5	160	[48]
Activated red mud	5	10	30	0.7	6	112	[45]
Iron oxide-activated red mud	6	0.4	25	1.5	6	1.05	[80]

**Table 5:** Effects of red mud on cadmium-contaminated soil.

Amendments	Remediation Time (d)	Cd Concentration in Red Mud (mg·kg^−1^)	Cd Concentration in Soil (mg·kg^−1^)	Soil pH before/after Red Mud Addition	Method of Cd Extraction	Change in Extractable Cd Concentration (%)	Reference
5% red mud	14	<10	4.1	7.2/7.5	1 M NH_4_NO_3_	−54	[87]
5% red mud	100	<10	4.1	7.2/7.5	1 M NH_4_NO_3_	−33	[87]
2% red mud	40	2.1	2.45	4.58/8.10	0.1 M Ca(NO_3_)_2_	−88	[42]
5% red mud	40	2.1	2.45	4.58/9.35	0.1 M Ca(NO_3_)_2_	−98	[42]
2% red mud	720	0.77	8.85	6.9/7	CH_3_COOHNH_4_	−1.0	[88]
5% red mud	720	0.77	8.85	6.9/7.2	CH_3_COOHNH_4_	−11	[88]
3% red mud	750	18	79	4.7/5.64	1 M NH_4_NO_3_	−24	[89]
5% red mud	750	18	79	4.7/5.64	1 M NH_4_NO_3_	−52	[89]
1% red mud	1095	4.1	5.6	4.9/5.7	1 M NH_4_NO3	−90	[83]
0.5% red mud	55	-	0.89	5.42/-	0.05 M EDTA-Na_2_	−11	[85]
1% red mud	55	-	0.89	5.42/-	0.05 M EDTA-Na_2_	−19	[85]
1% red mud	2191	4.0	13.0	5.5/7.6	1 M NH_4_NO_3_	−86	[86]
1% red mud	75	5.32	1.81	4.71/5.95	1 M MgCl_2_	−32	[48]
4% red mud	75	5.32	1.81	4.71/7.15	1 M MgCl_2_	−39	[48]
6% red mud	75	5.32	1.81	4.71/7.47	1 M MgCl_2_	−51	[48]

**Table 6:** Effects of red mud on cadmium accumulation in plants.

Crops	Amendments	Cd Concentration in Red Mud (mg·kg^−1^)	Culture Time (d)	Biomass Change (%)	Cd Concentration Change (%)	Plant Uptake Cd Change (%)	Reference
Green amaranth	1% red mud	1.5	90	-	-	−87	[94]
Red fescue	1% red mud	1.5	120	-	-	−38	[94]
Lettuce	2% red mud	2.1	-	+413	−83.5	−96.9	[36]
Lettuce	5% red mud	2.1	-	+131	−88.0	−95.0	[36]
Eulalia grass	2% red mud	2.12	90	-	−6	-	[95]
Bracken	2% red mud	2.12	90	-	−16	-	[95]
Pea	4% red mud	-	49	+77	−94	−89.1	[92]
Wheat	4% red mud	-	49	+446	−94	−67.8	[92]
Spinach	0.5% red mud	<0.01	37	+18.8	−46.7	−36.7	[91]
Tomato	0.5% red mud	<0.01	233	+6.96	−48.6	−45.0	[91]
Cabbage	0.5% red mud	<0.01	-	+8.70	−61.2	−57.9	[91]
Radish	0.5% red mud	<0.01	-	+22.3	−66.1	−58.4	[91]

Notes: Biomass change = (Aboveground biomass of crops after improvement—Aboveground biomass of crops before improvement)/(Aboveground biomass of crops before improvement) × 100%. Cd concentration Change = (Cd content in the aboveground parts of crops after improvement—Cd content in aboveground parts of crops before improvement)/(Cd content in aboveground parts of crops before improvement) × 100%. Plant uptake Cd Change = (Cd content in aboveground parts of crops after improvement × aboveground biomass—Cd content in aboveground parts of crops before improvement × aboveground biomass)/(Cd content in aboveground parts of crops before improvement × aboveground biomass) × 100%.

## Data Availability

Not applicable.
